# Novel Immunotherapeutics for the Treatment of Glioblastoma: The Last Decade of Research

**DOI:** 10.7759/cureus.2130

**Published:** 2018-01-30

**Authors:** Emaad M Khansur, Ashish H Shah, Kyle Lacy, Manish Kuchakulla, Ricardo J Komotar

**Affiliations:** 1 School of Medicine, University of Mississippi Medical Center; 2 Department of Neurological Surgery, University of Miami Miller School of Medicine; 3 Neurological Surgery, University of Miami Miller School of Medicine

**Keywords:** immunotherapy, glioblastoma, novel, outcome, clinical trial

## Abstract

Despite surgical resection and adjuvant chemoradiation, survival for glioblastoma remains poor. Because of the dismal prognosis, attention has shifted to alternative adjuvant treatment modalities. Although traditionally limited to systemic malignancies (melanoma, lung and colon cancer), the field of immunotherapy has recently identified glioblastoma as a potential target for new treatments. Anti-tumor vaccines (dendritic cell/heat shock), checkpoint inhibitors, chimeric T-cell receptors, and virotherapy all have been preliminarily trialed in glioblastoma patients with reasonable success and safety. Although there are limitations due to autoimmune reactions and immune escape, immunotherapeutics hold much promise in the future treatment paradigms for malignant glioma.

## Introduction and background

Glioblastoma is the most lethal primary central nervous system tumor with an incidence rate of 3.19 per 100,000 person-years, averaging around 13,000 cases diagnosed in the United States per year [1]. Over the last fifteen years, the treatment for glioblastoma multiforme (GBM) included maximal safe surgical resection with combination radiotherapy and adjuvant temozolomide chemotherapy [2]. Despite this treatment, the overall five-year survival still remains poor with an average survival of 14 months after initial diagnosis [2-4]. Although there have been significant advances in understanding the basic pathogenesis of GBM, median survival of patients has changed little in the last 25 years. Because of the dismal prognosis, attention has shifted to alternative adjuvant treatment modalities.

The idea of immunotherapy was first approached by William Coley over 120 years ago when he attempted to increase anti-tumor immune responses by administering bacterial toxins to reduce tumor recurrence. Although his initial attempts were unsuccessful, his research laid the groundwork for potential breakthroughs in the treatment of cancer. Recent research on cancer treatment has been focused on expanding Coley’s idea of immunotherapy by utilizing the immune system to target and effectively treat tumors by enhancing either the innate or adaptive immune system. With the Food and Drug Administration's (FDA) approval of Provenge (sipulecel-T, a dendritic cell-based therapy for prostate cancer) and Yerovry (ipilimumab for metastatic melanoma), research interest in immunotherapies in the treatment of cancer has expanded [5]. Current research on glioblastoma focuses on immunotherapy such as vaccines (dendritic cell/heat shock), checkpoint inhibitors, chimeric T-cell receptors, and immunogene therapy. See Table 1 for recent clinical trials for malignant glioma over the last five years. We will review the contemporary research on immunotherapeutics for glioblastoma.

**Table 1:** Recent immunotherapeutic clinical trial results over the last five years nGBM = newly diagnosed glioblastoma multiforme; rGBM = recurrent glioblastoma multiforme; PFS = progression free survival; OS = overall survival.

Name of trial	Type of therapy	Country	Patients	PFS (mo)	OS (mo)	Year
Phuphanich et al. [6].	Dendritic Cell	USA	17 nGBM 3 rGBM 1 brainstem glioma	16.9 nGBM	38.4 nGBM	2013
Sampson et al. [7].	Dendritic Cell	USA	22nGBM	15.2	23.6	2011
Mitchell et al. [8].	Dendritic Cell	USA	12nGBM	>27	>36.6	2015
Pellegatta et al. [9].	Dendritic Cell	Italy	15 rGBM	4.4	8.0	2013
Prins et al. [10].	Dendritic Cell	USA	15 nGBM 8 rGBM	-	35.9 nGBM 17.9 rGBM	2011
Vik-Mo et al. [11].	Dendritic Cell	Norway	7 nGBM	23.1	-	2013
Fadul et al. [12].	Dendritic Cell	USA	10 nGBM	9.5	28 months	2011
Bloch et al. [13].	Heat Shock	USA	41 rGBM	4.8	10.7	2014
Crane et al. [14].	Heat Shock	USA	12 rGBM	-	11.8	2013
Brown et al. [15].	Chimeric antigen T-Cell	USA	1 rGBM	7.5	-	2016
Ji et al.[16].	Adenovirus mutant thymidine kinase (ADV-TK)	China	53 rGBM	8.7	11.4	2015

## Review

Vaccine Therapy

Therapeutic cancer vaccines are designed to eradicate cancer cells by strengthening a patient's own immune response. These vaccines work by activating T-cells (CD4 and CD8) against specific tumor antigens and by inducing an anti-tumoral cellular response by using dendritic cells (DC) and heat shock proteins [17].

DC therapy

DC functions as antigen-presenting cells (APCs) by processing antigens peripherally and presenting them as antigenic peptides to the T lymphocytes [1]. The development of DC vaccines was predicated on the successful ex vivo culturing of mouse DC’s by Inaba, Steinman, and colleagues over 10 years ago. Current preparation of DC vaccines involves exposing the lysate of a patient’s tumor to the patient's autologous DCs, which are then treated with a differentiation factor such as GM-CSF. The primed APCs are then injected back into the patient with hopes of generating a T-cell response against the tumor [18]. Recently, DC vaccines have demonstrated some efficacy in improving outcomes for glioblastoma. In a recent systematic review, Bregy et al. demonstrated that autologous DC vaccination improved median OS in patients with newly-diagnosed and recurrent GBM compared to historical trends [19]. Beyond autologous tumor lysate, DC pulsed with specific tumor-associated antigens (TAA) from MAGE-1 and AIM-2 demonstrated prolonged survival in newly diagnosed GBM patients [6]. In order to improve the elicited immune response, Mitchell coupled DC vaccination with tetanus/diphtheria(Td) pre-conditioning. The Td toxoid served as a potent recall agent and improved DC migration to lymph nodes. The results of this study showed that there was a markedly enhanced bilateral DC migration that increased both the progression-free survival and overall survival when compared to DC only treated patients [8].

Aside from autologous DC vaccines, allogeneic DC vaccines have also been proposed. A study by Parney and Gustafson (2016) explored the benefits of adding DC therapy with concurrent temozolomide in patients with resected newly diagnosed glioblastoma. DCs were generated from the patient’s CD14+ monocytes, pulsed with allogeneic tumor lysate from two patient-derived GBM cell cultures, and given to patients during their temozolomide therapy. After vaccination, increased circulating tumor-associated antigen-specific CD8 T-cells were identified, demonstrating that allogenic tumor lysate vaccines are feasible and may generate a tumor antigen-specific immune response [20]. However, there are some inherent concerns of delivering allogeneic lysate to patients including vaccination rejection and lack of antigenic specificity; these concerns are partially mitigated by the fact that allogeneic lysate is derived from multiple cell lines and may be readily available after surgical resection.

In order to explore a new method of delivery, Sayour et al. (2016) examined the efficacy of delivering tumor-derived Ribonucleic acid (RNA) encapsulated in lipophilic nanoparticles in lieu of tumor lysate to systemically activate APCs for induction of therapeutic anti-tumor T-cell immunity. In preclinical murine GBM models, RNA nanoparticles were shown to exceed DCs in mediating anti-tumor activity. These formulations were also shown to be cost-effective and could be formulated expeditiously, providing rapid induction against GBM [21].

Heat shock protein (HSP) vaccines

The HSP function intracellularly to assemble and transport nascent proteins. HSPs also have a very critical role in the stress response to cellular insult and function by stabilizing proteins and preventing them from aggregating. Therefore, it is thought that they are transcriptionally upregulated in cancer due to increased translation of abnormal protein products. The two major HSP families that have been shown to be released by GBM exosomes are HSP70 and HSP90. The HSP70 family functions to inhibit cell stress-induced apoptotic pathways, facilitate protein folding, and guide protein transport across membranes. The HSP90 family is responsible for protein folding, protein stabilization, and loading onto Major Histocompatibility Complex (MHC) class I molecules. More importantly, HSP90 has been shown to be vital in tumor initiation and proliferation of signaling pathways. Thus, HSPs have been shown to have the potential to serve as a way to present tumor-specific antigens to elicit an antitumor immune response [22-25]. Tumor-derived HSPs and other proteins can be complexed together and serve as an antitumor vaccine in patients with glioblastoma. The advantage of these vaccines as compared to others is that HSPs are not targeted to a specific pre-defined antigen but instead to varying types of antigenic proteins upon vaccination, which serves to broadly target the intratumoral heterogeneity that is normally seen in GBM [22, 26-28].

HSP vaccination has generated a robust immune response as well. By binding autologous tumor-derived peptides to HSP-96, Crane et al demonstrated a significant peripheral immune response for the peptides bound to HSP-96 in 11/12 of the patients treated with recurrent GBM. Within this study, immune responders had a median survival of 47 weeks after surgery and vaccination, compared with 16 weeks for the single non-responder. Additionally, inflammatory cytokines (Interferon gamma, CD3 and CD8) were focally increased in the tumor-sites in the immune responder group, suggesting specific immune responses against autologous tumor derived peptides bound to HSP-96 [14]. Other studies seem to confirm the efficacy of HSP-96 vaccination. Bloch et al. (2014) reported a median overall survival of 42.6 weeks after HSP peptide complex-96 vaccination in patients with recurrent glioblastoma. Of note, 66% of patients in this study were lymphopenic prior to therapy, which is believed to have significantly impacted the anti-tumor immune response. Nevertheless, these studies demonstrate that the HSPPC-96 vaccination may be safe and deserve additional investigation [29].

After encouraging results from the previous phase II trials of HSPPC-96 on glioblastoma, a subsequent multi-institutional trial is being sponsored by Alliance for Clinical Trials of Oncology. This trial is examining whether HSPPC-96 can prolong overall survival in cases of recurrent GBM as an adjuvant therapeutic agent. The study consists of three arms: HSPPC-96 with concomitant bevacizumab, HSPPC-96 with the administration of bevacizumab at tumor progression, and bevacizumab alone. The primary measure of the study is OS with secondary outcomes, evaluating PFS and the safety and tolerability of the combination therapy [22].

Checkpoint Inhibitors

**Figure 1:**
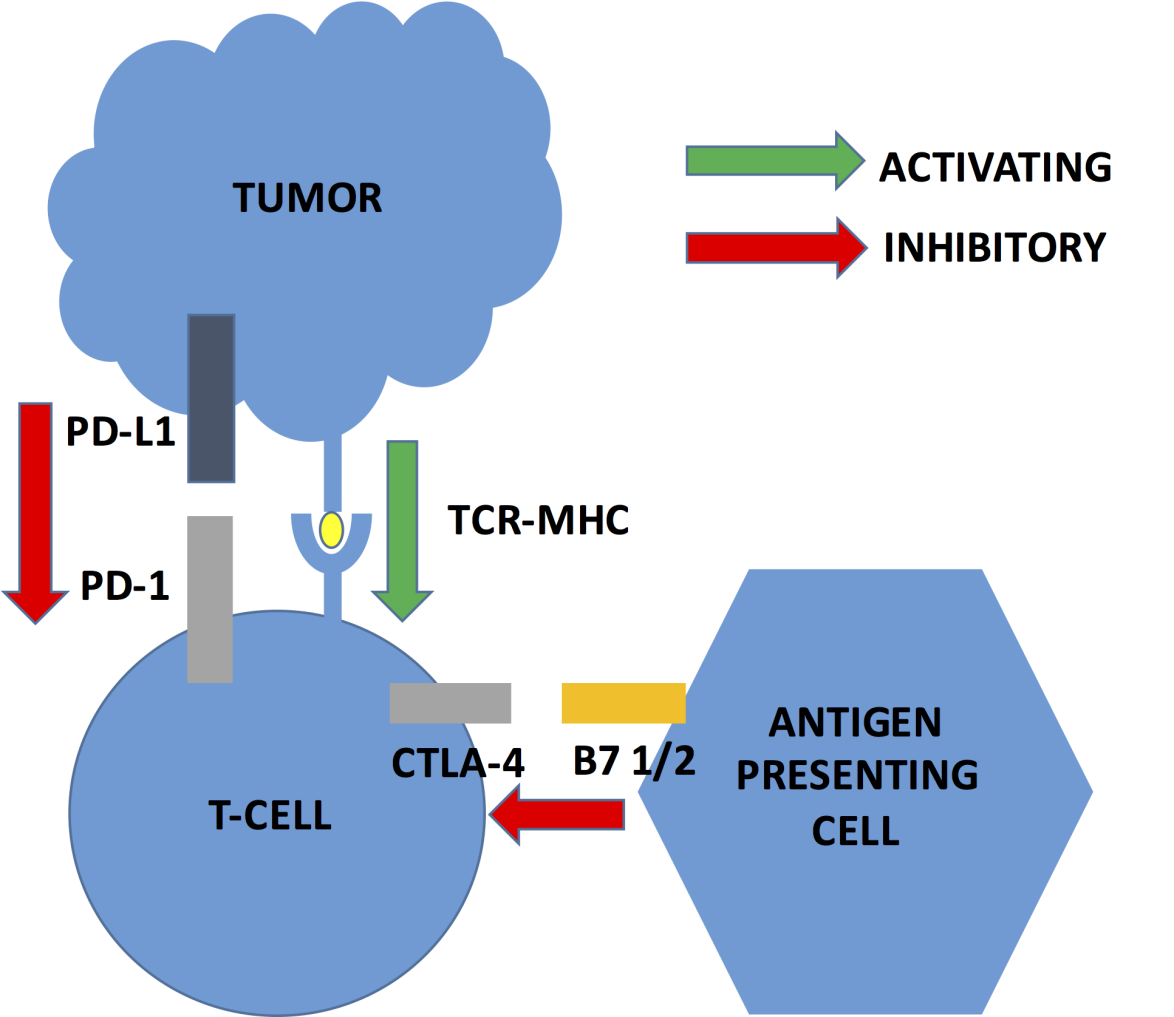
Major checkpoint inhibition pathways in cancer cells Programmed cell death protein and ligand (PD-1, PDL-1). Cytotoxic T-Lymphocyte Associated Antigen 4 (CTLA4).

Immune checkpoints are fundamental in the balance of self-tolerance and immunogenicity. Failed immune checkpoints impede immune responses in refractory cancers that are prone to T-cell anergy and toleragenicity. Programmed cell death protein and ligand (PD-1, PDL-1), metabolic enzymes (e.g., Arginase), and inhibitory immune pathways CTLA4 (Cytotoxic T-Lymphocyte Associated Antigen 4) have been hypothesized to play a role in immune tolerance. CTLA4, expressed on T-cells, regulates the extent of the T-cell immune response by impeding the CD28 T-cell stimulatory pathway [30]. In the clinical setting, CTLA4 blockade, through use of monoclonal antibodies, increases CD4 T-cell activity, and inhibits regulatory T-cell immunosuppression. In glioma mouse models, systemic blockade of PD-L1 demonstrated long-term survival with concurrent inhibition of regulatory T-cell activity [31]. Furthermore, PD-1 expressed more broadly than CTLA4 in the T-cells in the tumor microenvironment has been found to reduce T-cell activity in the peripheral tissues. Inhibition of PD-1 may augment the effector T-cells, antibody production and NK cell function [32-33]. In the clinical setting, PD-1 blockade demonstrated evidence of anti-tumor immunity in multiple cancers, with less immunotoxicity than with systemic CTLA4 blockade [34]. Additionally, inhibition of its ligand. PD-L1, on tumor cells, may also be a potential target for immunomodulation to prevent interaction with PD-1 receptors (see Figure 1 for major checkpoint inhibition pathways in cancer cells).

Several clinical studies assessing the importance of checkpoint inhibition in glioma have been performed. Berghoff et al. (2014) examined the expression of PDL1 in 135 glioblastoma specimens and noted diffuse or fibrillary PDL1 expression in 88% of samples from patients with newly diagnosed glioblastoma and in 72% of samples with recurrent glioblastoma. However, no correlation was found between PDL1 expression and survival [35]. Nevertheless, in animal models, activation of co-stimulatory receptors such as OX40 and blockade of co-inhibitory receptors such as PD1 and CTLA4 induced tumor regression and increased long-term survival [36]. Currently, several clinical trials are ongoing for assessment of monoclonal antibody checkpoint inhibitors (anti-PD-L1 and CTLA-4) for glioblastoma. (NCT02017717, NCT02617589, NCT02529072) Although preliminary results are not yet available, some issues with delivery and brain penetration of these systemic checkpoint inhibitors have been noted.

Chimeric T-cell Receptors (TCR)

Chimeric antigen receptors (CARs) are a diverse class of receptors that have been created by combining the variable region of an antibody with a T-cell-signaling molecule such as CD3. These newly created receptors are advantageous compared to the TCR-transduced T-cells. CARs have the ability to mimic endogenous TCR-mediated activation without the disadvantages of classical MCH restriction as the antigen recognition site is derived from an antibody. Additionally, these antibody directed-CARs can accommodate infinite antigenic diversity and nanomolar antigenic affinity. These receptors can also incorporate costimulator molecules such as CD28 and 4-1BB into the CD3 signaling domain which improves T-cell expansion, survival, and tumor lysis [37-39].

Several studies have demonstrated the safety and preliminary efficacy of this type of therapy in glioblastoma. Brown et al. examined the bioactivity and safety of IL13Ralpha2 redirected chimeric antigen receptor CD8 T-cells in the resection cavity of three patients, and noted transient immune-mediated anti-tumor responses in 2/3 patients with recurrent glioblastoma [40]. Other case reports of similar IL13Ralpha2-directed CAR conducted demonstrated tumor regression and immune responses after intrathecal therapy in patients with multifocal recurrent GBM [41]. Although clear improvements in survival was yet to be shown, this study provided promising results for potential phase 1 human clinical trials of IL13Ralpha2- specific CAR T-cell treatment for GBM.

Additional CAR-mediated treatments have also been investigated. O’ Rourke et al. (2016) utilized autologous T-cells re-directed to the EGFR variant III mutation in nine patients with recurrent glioblastoma. This study positively demonstrated a significant expansion of CART-EGFRvIII cells one week after infusion and tumor infiltration by activated CAR T-cells. This study showed that EGFRvIII-CAR therapy was safe without the evidence of off-target toxicity or cytokine release [41]. These studies helped establish a foundation that adoptive CAR T-cell therapy can be applied to the treatment of glioblastoma.

Viroimmunotherapy

The use of viruses to mediate gene immunotherapy in the treatment of tumors is a promising approach and has a wide variety of applications. Treatments can include transferring genes for inflammatory proteins to tumor cells, inhibition of immunosuppressing tumor genes, or transferring proinflammatory and tumor antigen genes to professional antigen presenting cells. Previous clinical trials have focused on conditional cytotoxicity and oncolytic viruses, which may induce a secondary immune response by generating foreign antigens and producing a pro-inflammatory immune beacon in tumor cells [42-43]. Several clinical trials using adenovirus, herpes simplex virus, replicating retroviruses have been conducted with preliminary results demonstrating survival benefit [16]. Many of these viral-based therapies utilize intraparenchymal convection-enhanced delivery methods to deliver the vector into the surgical cavity, and are now undergoing early phase I/II clinical trials [16, 44-46].

Pitfalls

While current research on the various treatments of glioblastoma has provided some encouraging results, each therapy has associated drawbacks. Clinical translation of DC vaccines has been modest due to limitations imposed by the source of antigens, poor DC maturation, tumor-mediated immunosuppression, and allergic encephalomyelitis [20]. The limitations of heat shock vaccines include the acquisition of adequate tissue for vaccine production and limited inclusion criteria (requiring near complete tumor resection) [22]. Both vaccination approaches are limited by the quality of the tumor lysate and the antigenic presentation; if tumor lysate is not representative of the heterogenic tumor or if the lysate does not induce antigen response, vaccination may not confer a benefit.

Other molecular immunotherapeutics are also limited in certain regards. The use of checkpoint inhibitors has potential adverse effects including severe autoimmune reactions which may include colitis, pneumonitis, hypophysitis, and hepatotoxicity [47]. Immune checkpoint inhibitors cannot function unless its target receptors (PD-1, CTLA-4) exist in the tumor microenvironment. Additionally, robust inhibition of the checkpoint pathway may result in uncontrolled systemic autoimmune reactions. In order to avoid this potential limitation, checkpoint inhibitors may need to be delivered intracranially to bypass systemic immune reactions. For chimeric antigen receptors, T-cell generation may limit treatment, and once delivered, antigens may be lost as the GBM adapts and modifies its antigen presentation by the glioblastoma [15].

## Conclusions

The burgeoning field of immunotherapy holds much promise for the treatment of glioblastoma. Novel biologics and pharmaceuticals are evolving treatment paradigms. The recent research in vaccine therapy, check-point inhibitors, chimeric antigen T-cell receptors, and viroimmuno therapy has provided an opportunity to supplement the current treatment of glioblastoma potentially, improving prognosis and overall survival for these patients. Although there are several barriers to an effective safe treatment, future larger prospective studies may help elucidate the role of immunotherapy in these patients.
